# Normal computed tomographic features and reference values for the coelomic cavity in pet parrots

**DOI:** 10.1186/s12917-016-0821-6

**Published:** 2016-09-05

**Authors:** Irene A. Veladiano, Tommaso Banzato, Luca Bellini, Alessandro Montani, Salvatore Catania, Alessandro Zotti

**Affiliations:** 1 Department of Animal Medicine, Production and Health, University of Padua, Viale dell’Università 16, Legnaro (PD), 35020 Italy; 2 Clinic for Exotic Animals, CVS, Rome, 00137 Italy; 3 Avian Medicine Laboratory, Istituto Zooprofilattico Sperimentale delle Venezie, Legnaro, 35020 Italy

**Keywords:** Monk parakeet, African grey parrot, Blue-and-gold macaw, Coelomic cavity, Computed tomography

## Abstract

**Background:**

The increasing popularity gained by pet birds over recent decades has highlighted the role of avian medicine and surgery in the global veterinary scenario; such a need for speciality avian medical practice reflects the rising expectation for high-standard diagnostic imaging procedures. The aim of this study is to provide an atlas of matched anatomical cross-sections and contrast-enhanced CT images of the coelomic cavity in three highly diffused psittacine species.

**Results:**

Contrast-enhanced computed tomographic studies of the coelomic cavity were performed in 5 blue-and-gold macaws, 4 African grey parrots and 6 monk parakeets by means of a 4-multidetector-row CT scanner. Both pre- and post-contrast scans were acquired. Anatomical reference cross-sections were obtained from 5 blue-and-gold macaw, 7 African grey parrot, and 9 monk parakeet cadavers. The specimens were stored in a −20 °C freezer until completely frozen and then sliced at 5-mm intervals by means of a band saw. All the slices were photographed on both sides. Individual anatomical structures were identified by means of the available literature. Pre- and post-contrast attenuation reference values for the main coelomic organs are reported in Hounsfield units (HU).

**Conclusions:**

The results provide an atlas of matched anatomical cross-sections and contrast-enhanced CT images of the coelomic cavity in three highly diffused psittacine species.

## Background

The increasing popularity gained by pet birds over recent decades has highlighted the role of avian medicine and surgery in the global veterinary scenario. The success of birds as pets is likely due both to the strong emotional connection linking such animals to their owners and to the high economical value of some species [1]. Among birds, psittacines have become one of the most diffused avian pet species; their appealing appearance, their deep interaction with the owner and the long lifespan expectation typical of some species represent the main reasons for their spread.

The increasing need for speciality avian medical procedures during recent decade reflects the rising expectation among owners for high-standard medical treatment for their pets.

Most parrots are so-called “stoic” animals; to avoid predation these animals evolved to mask signs of illness [2]. Accordingly, clinical signs are difficult to notice during routine examination, the role of ancillary diagnostic techniques becomes vital in the diagnosis of several different diseases.

Diagnostic imaging plays a key role in exotic pet medicine and several references aimed at standardizing normal features through different imaging techniques in exotic and wild mammals [3–8], reptiles [9–15], and birds [16–21] are currently available.

Some references describing the CT (computed tomographic) features of the coelomic cavity of avian species are currently available [16–21]. However, to the best of the authors’ knowledge, no specific references regarding the coelomic cavity in psittacines have yet been published. Moreover, little information is available regarding the use of contrast medium to enhance the visibility of individual coelomic organs in avian patients [22].

The aim of this study is to provide an atlas of matched anatomical cross-sections and contrast-enhanced CT images of the coelomic cavity in three highly diffused psittacine species (monk parakeet, African grey parrot, and blue-and-gold macaw). In addition, pre- and post-contrast attenuation reference values for the main coelomic organs are reported in Hounsfield units (HU).

## Methods

### Animals

Three different species of parrots were selected for the present study: blue-and-gold macaw (*Ara ararauna*), African grey parrot (*Pittacus erithacus*) and monk parakeet (*Myiopsitta monachus*). Five blue-and-gold macaws (Two males and Three females, mean weight 1000gr ± 14gr, mean length 86 cm ± 3.5 cm), Four African grey parrots (Three males and One females, mean weight 371gr ± 5gr, mean length 35 cm ± 1.5 cm) and Six monk parakeets (Three males and Three females, mean weight 130gr ± 2.5gr, mean length 28.5 cm ± 1 cm) presented to the University Veterinary Teaching Hospital of the University of Padua (Padua, Italy) were included in the study*. All the animals enrolled for the study were affected by head or limb pathologies (Seven head traumas and Eight leg fractures). Upon owner consent, whole body CT scans were performed.

### Anatomical procedures

The cadavers of Five adult-blue-and-gold macaws (Three males and Two females, mean weight 1003gr ± 13.5gr, mean length 85 cm ± 2 cm), Seven adult African grey parrots (Three males and Four females, mean weight 345gr ± 4.5, mean length 32 ± 2 cm), and Nine monk parakeets (Six males and Three females, mean weight 126gr ± 3gr, mean length 29 ± 0.5 cm) were used in this study. Within 24–36 h of death the cadavers were fixed in prone position on a plastic support and stored in a freezer (−20 °C) for 48 h. Consecutive 5-mm transverse slices were obtained by means of an electric band saw, from the inlet of the coelomic cavity to the cloaca. The slices were then numbered, cleaned with water and photographed on both sides. All the above animals died soon after hospitalization or were euthanized because of advanced medical conditions and their bodies were donated to the Veterinary Teaching Hospital of the University of Padua (Padua, Italy) or to the Clinic for Exotic Animals (Rome, Italy) by the owners.

### Imaging procedures

Computed tomography examinations were performed by means of a 4-multidetector-row CT scanner (Asteion S4, Toshiba Medical System, Amsterdam, NL). All live animals were anaesthetized with sevoflurane and oxygen administered via a facial mask, then intubated with an appropriate endotracheal tube; anaesthesia was maintained with sevoflurane carried by a mixture of medical air and oxygen. Computed tomographic studies were performed following a cranio-caudal direction with the animal in a prone position. Pre- and post-contrast sequences were acquired. Contrast medium (Optiray® 350 mg/ml, Covidien Spa, Italy) was injected in the right jugular vein with a 28-gauge needle at a dose of 660 mg/kg. The CT parameters were: helical acquisition mode, exposure time of 0.725 s, voltage of 120 kV, amperage of 150 mA, slice thickness of 1 mm.

The images were reconstructed with a soft tissue kernel and displayed in bone (window length 1000, window width 4000), pulmonary (window length −500, window width 1400) and abdomen (window length 40, window width 350) windows.

Attenuation was measured in Hounsfield units (HU) in the lungs, air sacs, liver, spleen, ventriculus, intestine and kidney in pre- and post-contrast scans using a commercially available DICOM processing software (Osirix, pixmeo SARL, Switzerland). Measurements were repeated three times using a region of interest of the same dimensions both in the pre and post-contrast images, and then averaged.

### Statistical analysis

Normality was graphically assessed by means of the Q-Q plot. Normally distributed data were reported as mean ± standard deviation whereas non-normally distributed variables were reported as median with the limits for the overall range. Differences among species were calculated through analysis of variance (ANOVA) in normally distributed data whereas differences were calculated utilizing the Kruskal-Wallis H Test in the non-normally distributed data.

## Results

**Fig. 1:**
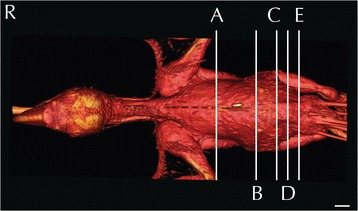
Three-dimensional reconstruction of the body of a blue-and-gold macaw. Lines A-E indicate the level of the matched cross-sections and CT images displayed in Figs. 2, 3, 4, 5, 6 and 7

**Fig. 2:**
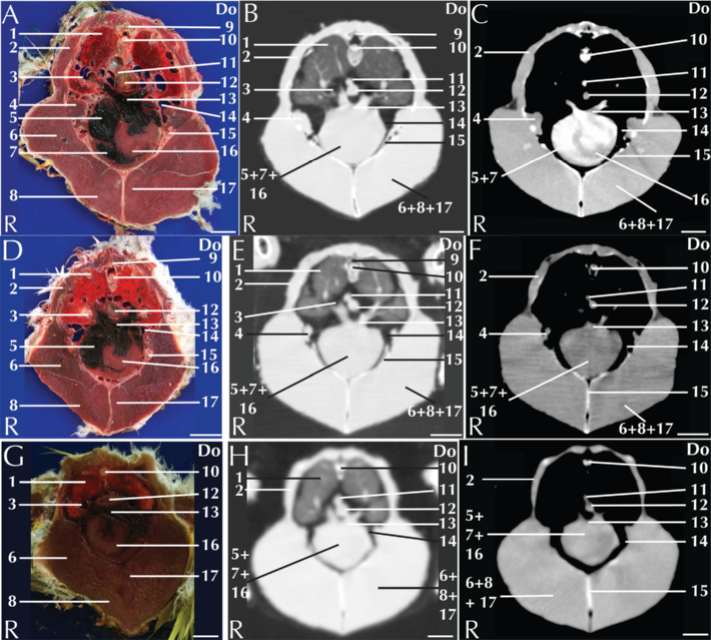
Matched cross-sections and CT images at the level of the heart corresponding to line A in Fig. 1. Matched cross-sections and CT images of blue-and-gold macaw (**a**-**c**), African grey parrot (**d**-**f**) and monk parakeet (**g**-**i**). The CT images have been reconstructed with a soft tissue kernel and displayed in pulmonary (**b**-**e**-**h**) and abdomen (**c**-**f**-**i**) window. Do is dorsal and R are right. Bar = 1 cm. 1. Lung; 2. Scapulohumeralis muscle; 3. Intrapulmonary primary bronchus; 4. Scapulohumeral caudal muscle; 5. Right atrium; 6. Pectoral muscle (thoracobrachialis portion); 7. Right ventricle; 8. Pectoral muscle (sternobrachialis portion); 9. Vertebra; 10. Spinal cord; 11. Aorta; 12. Oesophagus; 13. Pulmonary trunk; 14. Cranial thoracic air sac; 15. Carina sterni; 16. Cardiac muscle, left ventricle; 17. Supracoracoid muscle

**Fig. 3:**
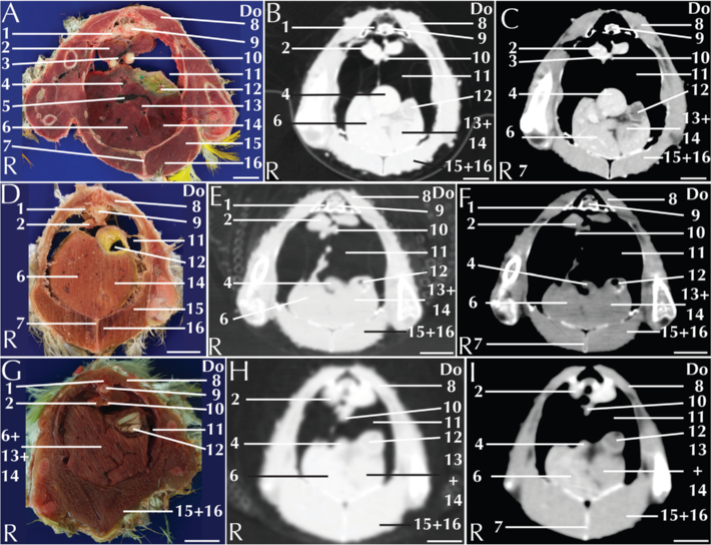
Matched cross-sections and CT images at the level of the liver corresponding to line B in Fig. 1. Matched cross-sections and CT images of blue-and-gold macaw (**a**-**c**), African grey parrot (**d**-**f**) and monk parakeet (**g**-**i**). The CT images have been reconstructed with a soft tissue kernel and displayed in pulmonary (**b**-**e**-**h**) and abdomen (**c**-**f**-**i**) window. Do is dorsal and R are right. Bar = 1 cm. 1. Vertebra; 2. Kidney (cranial lobe); 3. Common iliac vein; 4. Spleen; 5. Caudal vena cava; 6. Liver, right lobe; 7. Carina Sterni; 8. Longissimus dorsi muscle; 9. Spinal cord; 10. Gonads; 11. Air sac; 12. Proventriculus; 13. Liver, medial part of the left lobe; 14. Liver, lateral part of the left lobe; 15. Pectoral muscle; 16. Supracoracoid muscle

**Fig. 4:**
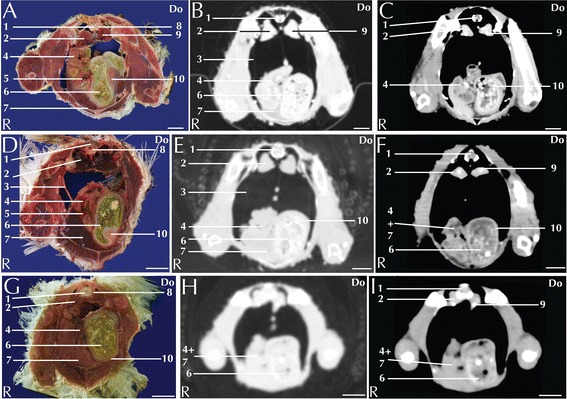
Matched cross-sections and CT images at the level of the ventriculus corresponding to line C in Fig. 1. Matched cross-sections and CT images of blue-and-gold macaw (**a**-**c**), African grey parrot (**d**-**f**) and monk parakeet (**g**-**i**). The CT images have been reconstructed with a soft tissue kernel and displayed in pulmonary (**b**-**e**-**h**) and abdomen (**c**-**f**-**i**) window. Do is dorsal and R are right. Bar = 1 cm. 1. Spinal cord; 2. Medial lobe of the right kidney; 3. Air sac wall; 4. Small intestine; 5. Pancreas. 6. Ingesta; 7. Liver; 8. Vertebra; 9. Left ureter; 10. Ventriculus, muscular wall

**Fig. 5:**
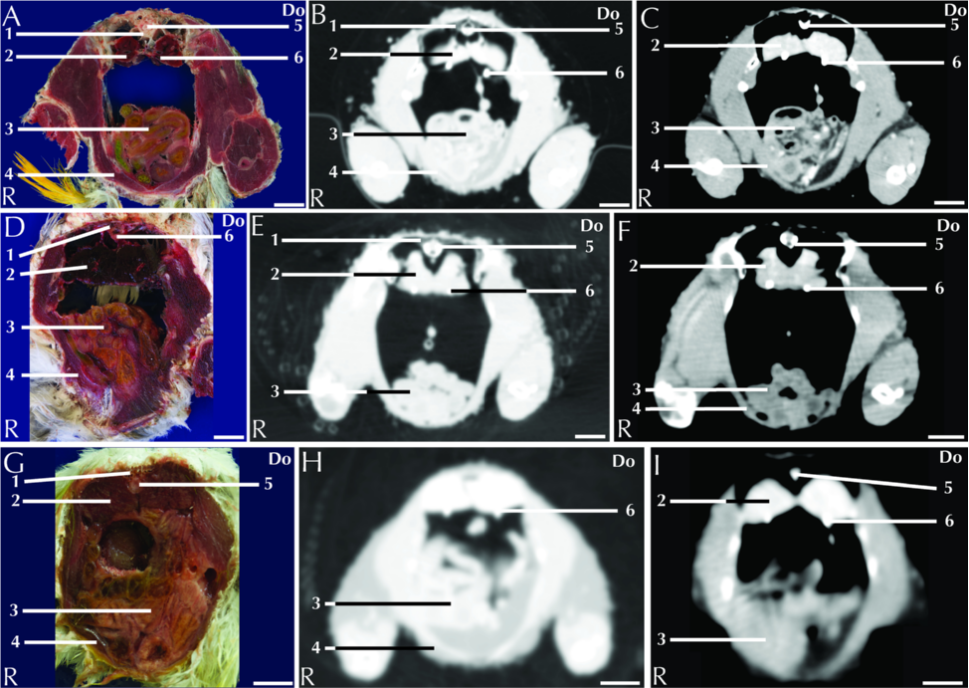
Matched cross-sections and CT images at the level of the kidneys corresponding to line D in Fig. 1. Matched cross-sections and CT images of blue-and-gold macaw (**a**-**c**), African grey parrot (**d**-**f**) and monk parakeet (**g**-**i**). The CT images have been reconstructed with a soft tissue kernel and displayed in pulmonary (**b**-**e**-**h**) and abdomen (**c**-**f**-**i**) window. Do is dorsal and R are right. Bar = 1 cm. 1. Vertebra; 2. Medial lobe of the right kidney; 3. Intestine; 4. Pectoral muscle (thoracic portion); 5. Spinal cord; 6. Left ureter

**Fig. 6:**
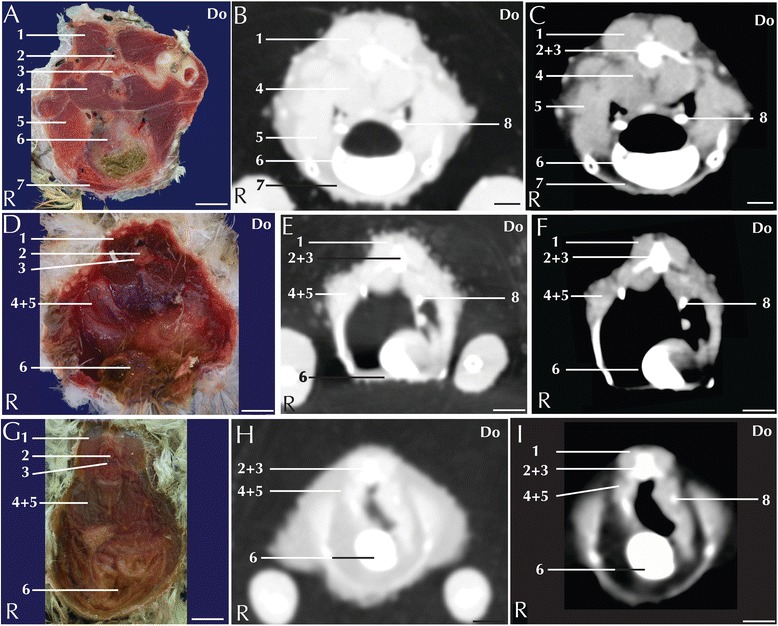
Matched cross-sections and CT images at the level of the cloaca corresponding to line E in Fig. 1. Matched cross-sections and CT images of blue-and-gold macaw (**a**-**c**), African grey parrot (**d**-**f**) and monk paraket (**g**-**i**). The CT images have been reconstructed with a soft tissue kernel and displayed in pulmonary (**b**-**e**-**h**) and abdomen (**c**-**f**-**i**) window. Do is dorsal and R are right. Bar = 1 cm. 1. Musculus levator caudae; 2. Spinal cord; 3. Vertebra; 4. Musculus levator cloacae; 5. Musculus transversus cloacae; 6. Cloaca; 7. Pectoral muscle (abdominal portion); 8. Left ureter

All the CT scans were performed on live animals and therefore a direct comparison with the anatomical images was not possible; nevertheless, visual inspections reveal a high correlation of both as shown in Figs. 1, 2, 3, 4, 5 and 6. Only post-contrast images are displayed. Individual anatomic structures were identified and labelled on the basis of the anatomical references [23–25], both in the anatomical cross-sections and in the corresponding CT images. All the main organs of the respiratory, digestive, urinary (including the ureters) and reproductive systems were visible both in the anatomical sections and in the corresponding CT images.

**Fig. 7:**
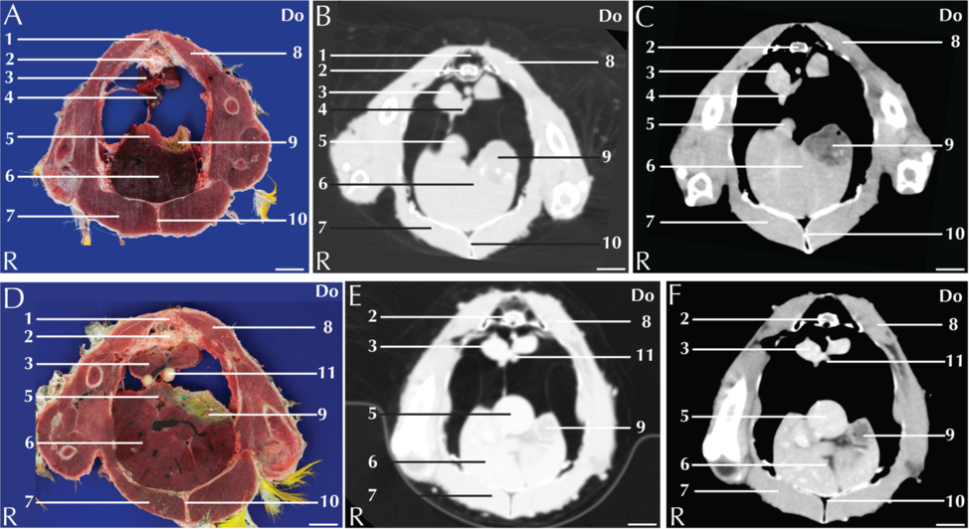
Matched cross-sections and CT images at the level of the gonads corresponding to line E in Fig. 1. Matched cross-sections and CT images of female blue-and-gold macaw (**a**-**c**), and male blue-and-gold macaw (**d**-**f**). The CT images have been reconstructed with a soft tissue kernel and displayed in pulmonary (**b**-**e**) and abdomen (**c**-**f**) window. Do is dorsal and R are right. Bar = 1 cm. 1. Vertebra; 2. Spinal cord; 3. Kidney; 4. Ovary; 5. Spleen; 6. Liver; 7. Pectoral muscle; 8. Longissimus dorsi muscle; 9. Proventriculus; 10. Coracoid bone; 11. Testicles

The approximate level of the reported matching cross-sections and CT images are shown in Fig. 1. The displayed matched cross-sectional images and CT scans shown in Figs. 2, 3, 4, 5, 6 and 7 are approximately at the same level in all the considered species, and the same structures are present in almost all the corresponding slices of different species.

All the considered HU values were non-normally distributed; hence the differences among species were calculated by means of the Kruskal-Wallis H test. As no statistically significant differences among species were evident for any organ, only descriptive statistics of the pooled data have been reported. Medians with the limits for the overall range of HU values, for the selected coelomic organs are reported in Table 1.

**Table 1:** Attenuation values measured in the CT scans of some selected coelomic organs, calculated in pre- and post-contrast CT scans. Pooled data from the considered species are reported

	Pre-contrast HU values	Post-contrast HU values
LIVER^a^	57.20 (33.14–104.66)	122.99 (86.85–172.83)
VENTRICULUS^a^	64.78 (36.19–91)	116.43 (92.37–143.13)
KIDNEY^a^	34.92 (12.39–74.86)	178.44 (151.52–378.97)
SPLEEN^a^	54.55 (23.11–81.3)	141.70 (123.90–181.95)
INTESTINE^a^	28.73 (10.8–59.53)	77.16 (32.92–102.09)
LUNG^a^	−589.47 (−677.26 - -478.48)	−579.97 (−690.50 - -385.73)
AIR SACS^a^	−957.84 (−969 - -929.32)	−982.06 (−1021 - -947.63)
TESTICLE^a^	22.20 (16.93–25.31)	47.60 (38.85–63.95)
OVARY^a^	15.94 (11.86–20.73)	80.97 (65.2–91.51)

^a^Data are reported as medians (with the limits of the overall range)

## Discussion

The coelomic cavity in the avian species is a single cavity with no further partitioning. Most of the coelomic cavity is filled by a very complex respiratory system that is composed of nine air sacs, six of which are in the coelomic cavity, plus two relatively small and non-expandable lungs.

The air sacs are air-filled structures covered by a thin pavimentous or cubical epithelium. This epithelium was clearly visible in CT scans of the blue-and-gold macaw and in the African grey parrot, but was not visible in monk parakeet (likely due to the small size of the animal) (Figs. 3b, e, h, 4b, e, h). The very limited vascularization of these structures brings differences that are undetectable between pre- and post-contrast scans [26].

The lungs are located cranio-dorsally in the coelomic cavity and are attached dorsally to vertebrae and ribs so that impressions of the ribs can be seen in isolated lung specimens [26]. These features were clearly visible in all the CT scans displayed in a pulmonary window (Fig. 2b, e, h). The trachea, the mainstem bronchi and the pulmonary arteries and veins could be clearly identified in all the examined species (Fig. 2b, e, h). No differences in the attenuation of the lung parenchyma after contrast medium injection could be noticed (Table 1). A high prevalence of respiratory diseases, both of bacterial and mycotic origin, is reported in pet parrots [26]. Although neoplastic pathologies are less frequent, a case of pulmonary adenocarcinoma in a blue-and-gold macaw has been reported [27]. It is also possible to find metastatic neoplasia in the lung parenchyma [28]. The use of CT in the diagnosis of respiratory diseases has been widely investigated in snakes [29] but, to the best of the authors’ knowledge, no references reporting the CT appearance of respiratory diseases in avian patients are currently available. However, thickened air sacs are a common pathological finding associated with respiratory disease [26–30].

The heart is located along the central axis of the body within the coelomic cavity in an indentation of the sternum, cranial to the liver (Fig. 2c, f, i). The four distinct chambers were visible in the CT images, displayed in an abdominal window in the blue-and-gold macaw (Fig. 2c). The aorta and pulmonary arteries were identified in all the examined species (Fig. 2c, f, i). It was possible to follow the aorta for its entire length. In birds, the aorta traces a curvy path turning to the right immediately after its ascending portion [31] (Fig. 2c, f, i).

The avian liver is a bi-lobed structure that lies on the sternum, wrapping cranially around the heart and dorsally around the lateral margin of the proventriculus. The right lobe is generally bigger than the left in parrots [32] (Fig. 3c, f, i). Most parrot species, including all the study species, lack gallbladder [32]. In the contrast-enhanced CT images displayed in an abdominal window, the main lobar veins were well visible within a homogeneous parenchyma (Figs. 3c, f, i, 4c, f, i). Pre- and post-contrast densities of the liver are reported in Table 1. The liver is a common target for metabolic lipidosis and for bacterial and viral disorders that affect the entire parenchyma or cause disseminated focal necrosis [30].

The avian stomach is composed of the proventriculus, a glandular portion located after the junction with the oesophagus and the ventriculus, a muscular portion located caudally in the digestive system [33]. The junction between the proventriculus and the ventriculus is called the isthmus. The proventriculus and the ventriculus are clearly visible in the left portion of the coelomic cavity (Figs. 3c, f, i, 4c, f, i). The ventriculus has a thick muscular layer that was clearly visible in all the examined species (Fig. 4c, f, i). In all the study subjects the ventriculus was filled with *ingesta* that appeared as a mixture of air and dense material with an ill-defined shape (Fig. 4c, f, i). It was possible to distinguish the grit as a highly attenuating element. Alteration of the stomach, specifically of the proventriculus, can be caused by infective pathologies. In particular, proventricular dilatation disease [34] and *Machrorabdus ornitogaster* infection could be considered as the most common infection related to alteration of the proventriculus shape. The isthmus is a common localization of *Machrorabdus ornitogaster* infection and gastric carcinoma [30].

Individual intestinal loops were visible only in the blue-and-gold macaw (Fig. 5c). Intestinal loop attenuation was difficult to evaluate both because of the presence of a mixture of *ingesta* and air and due to the limited dimensions of the intestinal wall. Nevertheless, the intestines appeared more attenuating in post-contrast scans (Table 1). Intestinal impaction, volvulus and intussusception have been described in avian patients [33]; although mycobacterial lesion (granulomas) can be relatively often recovered in the gut [35] there were no pathologies in our specimens.

The pancreas was located between the descending and ascending duodenal loops and was clearly visible in the anatomical section of blue-and-gold macaw and African grey parrot (Fig. 4a, d), but was not clearly distinguishable in the CT of any of the considered species.

The spleen was visible as a round structure lying to the left of the ventriculus (Fig. 3b, e, h, c, f, i). Pre- and post-contrast attenuation values of the spleen are reported in Table 1. Several infectious pathologies lead to increased overall dimensions of the spleen [30], but no reference have been made available to date.

Paired kidneys are located lateral to the spine and ventral to the pelvis. Although not clearly distinguishable in transversal CT images, multiplanar reconstructions (not shown in present paper) enabled to individually identify the three distinct lobes of the kidneys (Fig. 5c, f, i). A corticomedullary distinction was not evident. The ureters were visible, after contrast medium injection, in all the examined species and could be followed from the caudal pole of the kidney to the inlet in the cloaca (Fig. 6b, c, e, f, i). Pre- and post-contrast HU values are reported in Table 1. Renal pathologies are very common in avian patients; changes in shape and size of the renal parenchyma are associated with infections (acute or chronic), or chronic degenerative pathologies [30]. A case of renal tubule neoplasia has been reported in a channel-billed Toucan [36].

The gonads are located ventral to the cranial lobe of the kidney (Fig. 7c, f). When visible, the testicles appeared as moderately enhancing rounded paired structures whereas the ovaries appeared as single small, slightly elongated structures. In all the considered species, only the left ovary is functional. The gonads were clearly distinguishable only in sexually mature animals of the largest species (4 African grey parrots and 5 blue-and-gold macaw) (Fig. 7c, f). Although the gonads were clearly visible, the sex of the animal could not be determined in the smallest species (monk parakeet) because both the ovaries and the testicles had the same CT appearance. The oviduct was not visible in the anatomical images or CT scans. Pre- and post-contrast HU values are reported in Table 1. The reproductive organs of birds undergo cyclic atrophy and enlargement during the year in sexually mature animals [37]. Indeed, the male animals (2 african grey parrots, 1 monk parakeet) scanned during the reproductive season had remarkably larger testicles than the animals (2 blue-and-gold macaws, 1 African grey parrot, 2 monk parakeets) scanned during the non-reproductive period. Testicular and ovarian neoplasia are reported in avian patients [38, 39].

Although limited, the use of ultrasonography has been described in birds. The limited dimensions of the coupling sites and the presence of feathers act as limiting factors in the development of this imaging technique in avian patients. Some pathologic conditions, such as fluid accumulation, organ enlargement or displacement of the air sacs, enlarge the coupling site and therefore improve image quality. Ultrasonography is reported to be a useful diagnostic tool in the differentiation between cardiomegaly and hydropericardium in the investigation of masses involving the female reproductive tract and in the assessment of liver parenchymal changes. Gender determination through ultrasonography is possible only in mature animals during the breeding season; during the rest of the year and in immature animals the visibility of the gonads is hindered by the interposition of air sacs [40].

The lack of superimpositions and the fine anatomical resolution make CT the diagnostic imaging technique of choice when a pathology involving a coelomic organ is suspected. However, the clinician should also bear in mind both the potential risks associated with the general anaesthesia and the possible diagnostic information retrieved from a CT scan.

## Conclusions

The matched anatomical cross-sections and CT images presented in this study are a useful reference for the interpretation of CT examination of the blue-and-gold macaw, African grey parrot and monk parakeet.

All the images and descriptions presented in this paper relate only to the above-mentioned species. This atlas can be used as reference for other psittaccine species only if the clinician is aware of the anatomical and physiological differences occurring between the species under investigation and the species considered in this paper.

The complex anatomy of the coelomic cavity and the lack of intracoelomatic fat make the interpretation of plain radiographs often challenging in avian patients; on the other hand, the fine anatomical detail and the scope to evaluate coelomic organ vascularization make CT the gold standard diagnostic imaging modality if a pathology involving the coelomic cavity is suspected.

## Abbreviations

CT: Computed tomographic
